# Mean Warm Ischemia Time among Kidney Transplant Patients in a Tertiary Care Centre: A Descriptive Cross-sectional Study

**DOI:** 10.31729/jnma.8190

**Published:** 2023-06-30

**Authors:** Kalpana Kumari Shrestha, Pukar Chandra Shrestha, Swostik Pradhananga, Suraj Lama

**Affiliations:** 1 Department of Nephrology, Shahid Dharmabhakta National Transplant Center, Dudhpati, Bhaktapur, Nepal; 2 Department of Transplant Surgery, Shahid Dharmabhakta National Transplant Center, Dudhpati, Bhaktapur, Nepal

**Keywords:** *kidney transplantation*, *prevalence*, *warm ischemia*

## Abstract

**Introduction:**

In renal transplantation, warm ischemia time is the interval from the removal of a procured kidney from ice storage to initiating graft reperfusion. Successful kidney transplantation depends on warm ischemia time. The study aims to find the mean warm ischemia time among kidney transplant patients in a tertiary care centre.

**Methods:**

This descriptive cross-sectional study was conducted among kidney transplant patients in a tertiary care centre. Data from 15 December 2012 to 15 October 2022 were collected between

1 December 2022 to 4 January 2023 from the hospital records. Ethical approval was taken from the Nepal Health Research Council (Reference number: 1341). All first-time living-related kidney transplant recipients were included in the study. All the patients undergoing kidney transplants from brain-dead donors were excluded from the study. Convenience sampling method was used. Point estimate and 95% Confidence Interval were calculated.

**Results:**

Among 230 patients, the mean warm ischemia time was 35.45±7.35 min. The mean first warm ischemia time was 4.28±2.05 min and the mean second warm ischemia time was 31.27±7.04 min. The mean age of the recipients was 35.14±10.49 years (range 14-64), of which 173 (75.20%) were male and 57 (24.80%) were female.

**Conclusions:**

The mean warm ischemia time among kidney transplant patients in a tertiary care centre was similar to the studies done in similar settings.

## INTRODUCTION

Warm ischemia time (WIT) is the period from the removal of a procured kidney from storage to initiating graft reperfusion. There are two distinct phases of WIT. First WIT is the time a kidney remains at a body temperature after cutting off the blood perfusion by clamping a renal artery before it is cooled in ice storage and the second WIT is the period from the removal of an organ from cold storage to reperfusion. Successful kidney transplantation (KT) depends on factors like short WIT.^1^

Recent studies have identified rising WIT as a risk factor for poor early graft function.^2,3^ There is a substantial rise in WIT in the present era of laparoscopic nephrectomy compared to open nephrectomy.^4^ In Nepal, studies on WIT have not been carried out yet as Nepal has merely stepped into the second decade of a KT. There is a substantial knowledge gap related to KT in Nepal.

The aim of this study was to find out the mean warm ischemia time among kidney transplant patients in a tertiary care centre.

## METHODS

This descriptive cross-sectional study was conducted in the Department of Transplant Surgery in Shahid Dharmabhakta National Transplant Center, Dudhpati, Bhaktapur, Nepal from where data from was collected from the hospital record section. Data from 15 December 2012 to 15 October 2022 were collected between 1 December 2022 to 4 January 2023 from the hospital records. The ethical approval was taken from the Nepal Health Research Council (Reference number: 1341). All first-time living-related kidney transplant recipients were included in the study. All the patients undergoing kidney transplants from brain-dead donors were excluded from the study. Convenience sampling method was used. The sample size was calculated using the following formula:


$$\begin{array}{ll} \text{n} & ={\text{Z}}^{2}\times \frac{{\text{σ}}^{\text{2}}}{{\text{e}}^{\text{2}}}\\ & =1.96^{2}\times \frac{37.9^{2}}{5^{2}}\\ & =221 \end{array}$$

Where,

n = minimum required sample size
Z = 1.96 at 95% Confidence Interval (CI)
σ = standard deviation is taken as 37.9 from published literature^5^
e = margin of error, 5%

The minimum required sample size was 221. However, the final sample size taken was 230. The patient's demographic and clinical characteristics were recorded as per the proforma. Total WIT was grouped as short (<30 mins), intermediate (30-45 mins), and prolonged (>45 mins).^6^

Data were entered in Microsoft Excel 2016 and analysed using IBM Statistics SPSS 18.0. Point estimate and 95% CI were calculated.

## RESULTS

Among 230 patients, the mean WIT was 35.45±7.35 mins. The mean first WIT was 4.28±2.05 mins and the mean second WIT was 31.27±7.04 mins. The mean age of the recipients was 35.14±10.49 years (range 14-64), of which 173 (75.20%) were male and 57 (24.80%) were female. The mean hospital stay was 14.57±8.25 days. Only 15 (6.50%) of the cases had prolonged WIT (Table 1).

**Table 1:** Warm ischemia time (n= 230).

Warm ischemia time (mins)	Mean+SD
First WIT	4.28±2.05
Second WIT	31.27±7.04

Among 230 patients, 161(70%) patients hadintermediate warm ischemia time (Table 2).

**Table 2:** Warm ischemia time (n= 230).

Warm ischemia time	n (%)
Short WIT	54 (23.48)
Intermediate WIT	161 (70)
Prolonged WIT	15 (6.52)

The mean age of patients was 35.14±10.49 years. Among 230 patients, 173 (75.20%) were male (Table 3).

**Table 3:** Socio-demographic characteristics (n= 230).

Characteristics	n (%)
**Gender**	
Male	173 (75.20)
Female	57 (24.80)
**Ethnicity**	
Brahmin/Chhetri	88 (38.26)
Terai/Madheshi	14 (6.08)
Dalit	17 (7.39)
Newar	37 (16.08)
Janajati	62 (26.96)
Muslim	7 (3.04)
Others	5 (2.17)

## DISCUSSION

In our study, we defined total WIT as the summation of first WIT and second WIT. Among 230 patients, the mean WIT was 35.45±7.35 mins. A study in Indonesia showed a total WIT of 36.56 mins.^7^ In our study, 161 (70%) of cases had intermediate WIT, followed by 54 (23.5%) with short WIT and 15 (6.5%) of cases had prolonged WIT. Similarly, a study in China showed first WIT of 3.7±3.3 mins in living-donor renal transplant patients.^8^ A multicenter study in Canada showed median total WIT of 35 mins (Q1-Q3: 27-45 mins).^9^ The WITs in these studies are similar to our study findings.

Many studies showed that recipients' body mass index, multiple renal arteries, and right donor kidney are the three main risk factors of prolonged (>40 mins) WIT.^10^ However, we cannot associate these factors as the cause of the prolonged WIT at our centre due to the descriptive nature of our study. Many factors determine the early outcome of a living donor kidney transplant (LDKT). The impact of cold ischemia time (CIT) has been studied widely.^11^ However, very less is known about the impact of WIT and its impact on early outcomes such as delayed graft function (DGF). The pathophysiology behind this is that as the kidney is removed from the cold storage, the temperature of the allograft can rise to 15°C within 20 mins. This temperature is a metabolic threshold at which cellular metabolism increases, likely damaging the transplanted allograft by ischemia-reperfusion injury.^12^ This can be manifested clinically as DGF which requires a longer hospital stay for medical management. Interestingly, there is conflicting evidence of the relationship between prolonged WIT and Length of hospital stay (LOS). In a study, it was found that WIT greater than 29 min increased the risk of DGF by 3.5 times and every added 5 min resulted in 1 extra day in the hospital.^3^ On the contrary, another study showed no correlation between WIT and LOS.^12^ For future studies, it will be beneficial to measure the association between WIT on LOS as the optimal LOS can help in minimizing health resources and costs without compromising quality care.

Globally, WIT has become an interesting research topic due to the sudden increment in procedures like partial nephrectomy for cancer treatment.^13,14^ However, in our case, WIT became an area of research due to the conduction of a high volume of renal transplantation surgery. In Nepal, the KT procedure is relatively at a younger stage, and study related to KT is fewer. Therefore, there is a big knowledge gap related to KT; up to now, there is no study of WIT during KT in Nepal. In this study, we tried to show the WIT in renal transplantation procedures.

There are a few limitations in this study. This is a descriptive study, therefore, the analytical parameters could not be evaluated. Also, the study is based on a single centre and the findings of this study cannot be generalized to the general populations across the nation unless similar findings are seen in other transplant centres.

## CONCLUSIONS

The warm ischemia time of renal transplant procedure at our centre was found to be similar to studies done in other international renal transplant centres. The majority of the patients have short to intermediate warm ischemia time.

## References

[1] Campbell S, Pilmore H, Gracey D, Mulley W, Russell C, McTaggart S (2013). KHA-CARI guideline: recipient assessment for transplantation.. Nephrology (Carlton)..

[2] Ferede AA, Walsh AL, Davis NF, Smyth G, Mohan P, Power R (2020). Warm ischemia time at vascular anastomosis is an independent predictor for delayed graft function in kidney transplant recipients.. Exp Clin Transplant..

[3] Marzouk K, Lawen J, Alwayn I, Kiberd BA (2013). The impact of vascular anastomosis time on early kidney transplant outcomes.. Transplant Res..

[4] Thomson DA, Muller E, Kahn D (2014). Laparoscopic kidney donation - giving in the best way possible.. S Afr J Surg..

[5] Gures N, Gurluler E, Berber I, Karayagiz AH, Kemik O, Sumer A (2013). Comparison of the right and left laparoscopic live donor nephrectomies: a clinical case load.. Eur Rev Med Pharmacol Sci..

[6] Hellegering J, Visser J, Kloke HJ, D'Ancona FC, Hoitsma AJ, van der Vliet JA (2012). Deleterious influence of prolonged warm ischemia in living donor kidney transplantation.. Transplant Proc..

[7] Nugroho EA, Hidayat A, Hidayat AT (2019). Correlation of warm and cold ischemic time to graft function in kidney transplant: a single centre report.. Open Urol Nephrol J..

[8] Zhang Z, Liu Z, Shi B (2021). Global perspective on kidney transplantation: China.. Kidney360..

[9] Tennankore KK, Kim SJ, Alwayn IP, Kiberd BA (2016). Prolonged warm ischemia time is associated with graft failure and mortality after kidney transplantation.. Kidney Int..

[10] Hellegering J, Visser J, Kloke HJ, D'Ancona FC, Hoitsma AJ, van der Vliet JA (2012). Deleterious influence of prolonged warm ischemia in living donor kidney transplantation.. Transplant Proc..

[11] Ojo AO, Wolfe RA, Held PJ, Port FK, Schmouder RL (1997). Delayed graft function: risk factors and implications for renal allograft survival.. Transplantation..

[12] Ferede AA, Walsh AL, Davis NF, Smyth G, Mohan P, Power R (2020). Warm ischemia time at vascular anastomosis is an independent predictor for delayed graft function in kidney transplant recipients.. Exp Clin Transplant..

[13] Becker F, Van Poppel H, Hakenberg OW, Stief C, Gill I, Guazzoni G (2009). Assessing the impact of ischaemia time during partial nephrectomy.. Eur Urol..

[14] Patel AR, Eggener SE (2011). Warm ischemia less than 30 mins is not necessarily safe during partial nephrectomy: every min matters.. Urol Oncol..

